# Cues to Androgens and Quality in Male Gibbon Songs

**DOI:** 10.1371/journal.pone.0082748

**Published:** 2013-12-18

**Authors:** Claudia Barelli, Roger Mundry, Michael Heistermann, Kurt Hammerschmidt

**Affiliations:** 1 Sezione di Biodiversità Tropicale, MUSE - Museo delle Scienze, Trento, Italy; 2 Reproductive Biology Unit, German Primate Center, Göttingen, Germany; 3 Department of Primatology and Department of Developmental and Comparative Psychology, Max Planck Institute for Evolutionary Anthropology, Leipzig, Germany; 4 Endocrinology Laboratory, German Primate Center, Göttingen, Germany; 5 Cognitive Ethology Laboratory, German Primate Center, Göttingen, Germany; University of Sussex, United Kingdom

## Abstract

Animal vocal signals may provide information about senders and mediate important social interactions like sexual competition, territory maintenance and mate selection. Hence, it is important to understand whether vocal signals provide accurate information about animal attributes or status. Gibbons are non-human primates that produce loud, distinctive and melodious vocalizations resembling more those of birds than of other non-human primates. Wild gibbons are characterized by flexibility in social organization (i.e., pairs and multimale units) as well as in mating system (i.e., monogamy and polyandry). Such features make them a suitable model to investigate whether the physiology (hormonal status) and socio-demographic features find their correspondence in the structure of their songs. By combining male solo song recordings, endocrine outputs using non-invasive fecal androgen measures and behavioral observations, we studied 14 groups (10 pair-living, 4 multimale) of wild white-handed gibbons (*Hylobates lar*) residing at Khao Yai National Park, Thailand. We collected a total of 322 fecal samples and recorded 48 songs from 18 adult animals. Our results confirmed inter-individuality in male gibbon songs, and showed a clear correlation between androgen levels and song structures. Gibbons with higher androgen levels produced calls having higher pitch, and similarly adult individuals produced longer calls than senior males. Thus, it is plausible that gibbon vocalizations provide receivers with information about singers' attributes.

## Introduction

Vocalizations are assumed to provide information about senders, including their identity [1]–[3], rank [4], [5], age, sex or size [6], [7]. Given the importance of vocal signals in mediating crucial social interactions (i.e., sexual competition, territorial maintenance, partner or parent/young recognition [8]) in birds, anurans and mammals vocal signals (e.g., duration and fundamental frequency) should provide accurate information about animal attributes or status. They may also convey honest information because only individuals in better condition should be more capable to afford any associated costs with signal production.

In birds, hormones play a central role in singing behavior. Studies on birds of the temperate-zone have shown that incidence and complexity of songs are closely related to changes in androgen (i.e. testosterone) levels [9], [10]. These behavioral changes find their correlates in neuronal structural changes of birds' brains, which is one of the most spectacular examples of neuroplasticity [11]. Apart from the endocrine component, age, social status and reproductive success have also been reported to be correlated with vocal performance [12]–[14].

Similar to birds, mammals' vocal displays are also often mediated by androgens [15], [16]. These hormones affect vocalizations through actions on motivational centers and vocal motor pathways in the central nervous system [16] or via modulation of peripheral structures involved in signal production. For example, changes in androgens may influence the pitch of vocalizations in giant pandas (*Ailuropoda melaneuca*: [17]) or affect the trill rate in Neotropical singing mice (genus *Scotinomys*: [18]). The influence of androgens on the structure of mammalian vocalizations and pronunciation [19], [20] is relatively well documented for humans [20]–[22], but in non-human primates no direct evidence has yet been found (baboons: [23], [24]). Age, possibly due to age-related vocal fold growth, as well as social status may have an influence on fundamental frequency (F0; e.g., humans [25], [26]; baboons [4], [6], [27]; chimpanzees [28]). However, there is evidence for a high level of independence between F0 and age (e.g., humans [29]; non-human primates [30]).

Based on the hypothesis that vocal fold morphology is not exclusively relevant to vocal differences [31], [32], vocal folds' mechanical properties may shape specific vocal signals such as F0 [32], [33]. Moreover, androgen levels, as well as other features (e.g., age, social status), may directly act on laryngeal muscles and connective tissue of vocal folds by constantly remodeling them [34], [35]. Changes in hormonal levels have an obvious physiological explanation during adolescence when androgen variations have a permanent impact in the length or tension of the vocal folds [32]. However, after maturation, androgens may still vary and fluctuate in a reversible manner. Androgen changes may thus have also a psychological component affecting the vocal production which an individual may use as part of a social interaction strategy (e.g., humans [21]).

All in all, it is plausible that inter-individual variation of vocal signals and their related components in anatomy, physiology and behavior can lead to vocal differences between individuals and identify individuals, which in turn may convey information to different types of receivers, for example, to potential competitors (humans [26]; red deer [36]; domestic dogs [37]) or to potential mates (humans [38]–[40]; other mammals [41]).

Among non-human primates, gibbons are of particular interest since they produce distinctive vocalizations (‘song’) which are species-specific [42], [43]. Male songs (‘solos’), which occur in addition to gibbons' well known female-male duets, are loud enough to be heard up to a kilometer away [44] and can last for up to 4 hours [45]. Gibbon solo songs may function in one or more of several ways, including home range defense against neighboring males and solitary conspecific strangers as well as communicating with candidate mates and strengthening pair bonds [44], [46]–[50]. As a consequence, gibbon solo songs have developed clear adaptations to improve long-distance transmission [51]–[54] making them very different from other non-human primate vocalizations, but resembling those of rainforest birds.

Despite the existence of some basic information about male gibbon vocalizations, to date it is completely unknown whether individual attributes of the caller are encoded in the acoustic structure of their songs. Thus, we investigated the wild white-handed gibbon (*Hylobates lar*) population residing at Khao Yai National Park, Thailand, which is characterized by flexibility in both social organization (i.e., single-male/single-female groups and groups with one female and more than one adult male [55], [56]) and mating system (monogamy and polyandry occur, as well as extra-pair copulations and conceptions [55], [57], [58]). Moreover, a recent study has revealed a close association between androgen levels and social organization, with higher androgen concentrations being found in males living in pairs rather than those living in groups with more than one male [59]. All these features make this population particularly suitable to examine whether male solo songs' structure and its specific acoustic parameters are related to physiological status (i.e., androgen levels), as well as to socio-demographic features (i.e., social status or age). We first assess (i) inter-individual differences in song structure between gibbon males. Later, we combine male solo song recordings and endocrine outputs using non-invasive fecal androgen measures and behavioral observations. We describe and identify the relationship between song structure (e.g., F0 and duration) and (ii) androgen levels, (iii) social status, and (iv) age. Specifically, considering the spectral domain, we expect to find some sort of association between androgens and pitch, whereas the direction is difficult to predict. Regarding the temporal domain, a positive relationship between call duration and androgens would be expected. Considering the different social units (i.e., pairs and unifemale/multimale units) and previous findings that males living in pairs showed higher androgen levels than those living in groups with more than one male, we also predict that pitch varies between different types of males (i.e., the only male in pairs, primary or secondary males in unifemale/multimale units).

## Materials and Methods

### Ethics Statement

All fieldwork was performed without direct contact or interaction with the individuals and under research/collecting permit number 2.3/2210 to CB, issued by the National Research Council of Thailand (NRCT) and the National Park, Wildlife and Plant Conservation Department (DNP) of Thailand. Fecal material was imported into Germany under the permit V3/19j 06.23 issued from the Hessian Ministry for Environment, Energy, Agriculture and Consumerism. A CITES permit was not required as animal excretory products (urine, feces) do not fall under CITES regulations.

### Gibbon population and study site

At time of data collection, the study population residing in the Mo Singto-Klong E-Tau area, located in the central part of the Khao Yai National Park, Thailand (2,168 km^2^; 14°26′ N, 101°22′ E; ∼150 km NE of Bangkok), hosted a total of 24 males living in 14 well-known habituated groups. Khao Yai National Park consists of a sandstone plateau ranging between 600 m and 1000 m above sea level and covered by seasonal evergreen forest [60]. The study area is a continuous forest on a hilly terrain of 8.5 km^2^ which hosts approximately 15.9 individuals of white-handed gibbons per km^2^ [57]. Group size ranges between two and six individuals, usually consisting of a mated pair with their putative offspring, or multimale units with a single breeding female and two or more sexually mature males [57], [61]. Group home ranges tend to be of approximately 400–500 meters in diameter. Out of the 24 males available, we were successful in recording solo songs from 18 animals (three males performed only duets, while for 3 others acoustic data were not available; Table 1). Among those, 14 males were considered adults, both primary and secondary males (for definition see below), and four were classified as subadults, which were fully grown males but still residing in their natal group (Table 1).

**Table 1:** Khao Yai white-handed gibbons present during the study period.

Group	Female	Primary male	Secondary male	Subadult*
A	Andromeda	Chuū‡	Cassius II.	
			Christopher	
B	Baak	Chet	–	
C	Cassandra	Chana	–	
D	Daow	David	–	Dodo
E	Emanuelle**	Fearless	–	
H	Hannah	Felix	–	Henry
J	Jojo	Lung°	–	
M	Rung	Chikyu‡	–	
N	Hima	Nithat	Claude	
			Noi	
NOS	Nasima	Nissan	Nostradamus	Nosi
R	Brit	Elias°	–	
S	Sophi	Shaft	Samran°	
T	Brenda	Amadeus‡	–	
W	Wolga	Wotan	–	William
*Total*		*14*	*6*	*4*

* : individual males for which only descriptive analysis is available (*see* Table 4);

** : *H. pileatus* female coupled with a *lar* male for several years;

° : animals excluded from analysis (N = 3) because of missing samples;

‡ : animals excluded from analysis (N = 3) because no male solo songs were recorded (only duets were available).

Only adult males were considered in the analysis.

### Social organization, social status and age class

Due to the flexible social organization of the Khao Yai gibbons [55], [56], during the study period 10 of the 14 focal groups consisted of a single adult female, a single adult male and up to three offspring, reflecting a pair-living social organization (cf. [62]). The remaining four groups were considered unifemale/multimale units, with a single adult female and at least two adult males unrelated to the resident female (Table 1; [55], [58], [59]).

We classified all study males as either 'primary' or 'secondary' [54], [57]. Primary males were the only males in the pair-living groups or those males in unifemale/multimale groups [55] which predominantly engaged in singing duets with the adult female and performed the majority of copulations with them (*N* = 9). All remaining males in unifemale/multimale units, who rarely sang or copulated with the group's adult female [55] were considered as secondary (*N* = 5).

To test predictions about the relationship between age and acoustic features, we classified males into two age categories: adult (8–25 years of age) and senior (exceeding 25 years of age [59]). Since a few males were mature in age (i.e., falling in the adult category) but still residing in their natal group (i.e., falling in the subadult category), we decided to exclude them from analysis and present only descriptive information.

### Fecal sampling and androgen analysis

To examine the correlation between male androgen status and acoustic parameters, we determined fecal androgen metabolite levels for each adult male sampled and related them to their vocal structure. While recording vocalizations, three teams of field assistants collected fecal samples from target animals (all individuals are well known since decades and recognized by individual natural markers [57]) for endocrine analysis of 18 males (including males classified as subadults). Each team followed four to five of the 14 study groups from dawn to dusk (mean observation time: 8 h/day) for three months each. All samples were collected between 6:30 and 14:00 directly following defecation (for details *see* [59]). A total of 322 samples uncontaminated by urine (15 samples per male; range 5–29) were collected, kept on ice until arrival at the field station and then stored frozen until transport to the endocrinology laboratory of the German Primate Center (DPZ) for measurement of androgen content. Specifically, samples were analyzed for immunoreactive epiandrosterone (EA), a major metabolite of testosterone in primate feces [63]–[65], using an enzyme immunoassay (EIA) recently validated for monitoring androgen output in the white-handed gibbon [59]. Fecal extractions and assay procedures were carried out as previously described [59], [63]. Intra-assay coefficients of variation (CV) for a high- and low-concentrated quality control were 7.1% and 8.5%, respectively. Corresponding figures for inter-assay CVs were 12.7% and 15.9%.

### Acoustic recordings and analysis

We collected most of the acoustic data within a period of six months, between October 2008 and March 2009, with a second set of recordings being undertaken between April and May 2010. We recorded gibbon vocalizations *ad libitum* using a Sennheiser directional microphone (K6 power module and ME66 recording head with MZW66 pro windscreen) and two Marantz solid state recorders (PMD 660 and 670). We daily followed focal groups from dawn till dusk (average 8 hrs/day) and, whenever a male started singing, we recorded his vocalization within a distance of 5–20 meters. Information regarding subject identity and context was always spoken onto the tape or noted down into spreadsheets. Sounds were recorded in mono format with 16-bit resolution and 44.1-kHz sampling rate.

**Figure 1:**
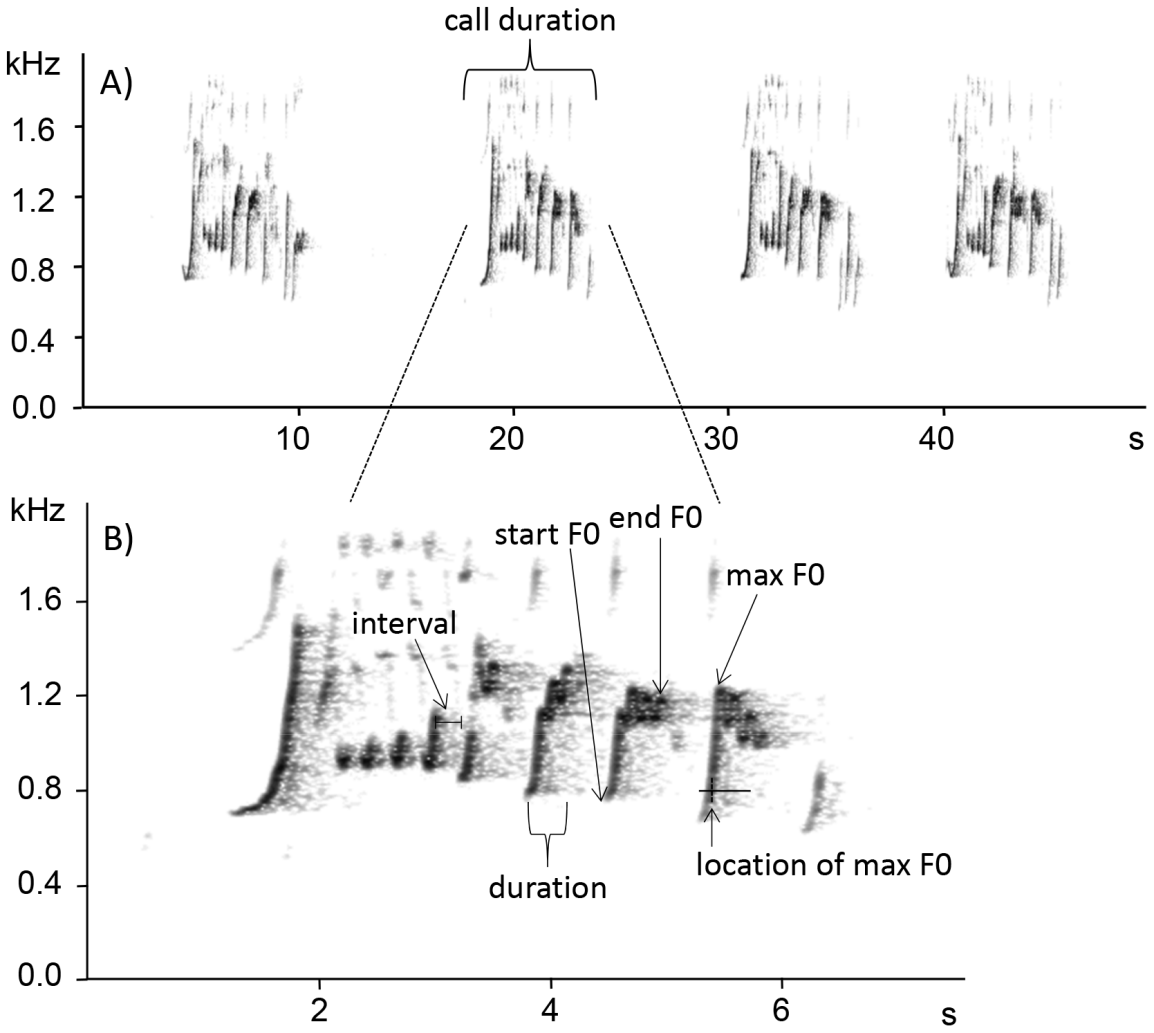
Example of male gibbon solo song's spectrogram composed by four calls (A) and enlargement of a single call (B) illustrating each element and its estimated acoustic parameters (i.e., interval between elements, element duration, start F0, end F0, max F0, mean F0 and location of max F0).

Vocalizations were characterized by a number of structural and temporal parameters. We included temporal measurements because changes in androgen levels could also lead to motivational changes which likely influence the temporal structure of primate vocalization. We defined as 'element' the single note uttered by a singing individual, while a sequence of undefined number of elements, separated by a short interval of time between each other, was classified as 'call'. Combinations of call sequences identified male 'song' for each individual gibbon (Fig. 1). To obtain an adequate frequency resolution, we down-sampled files from 44.1 kHz to 8 kHz. By using SASLab Pro 5.1 (Avisoft Bioacoustics, Berlin, Germany), we estimated several parameters describing the frequency modulation of F0 which in gibbons is the frequency with the highest amplitude [66], [67]. We used the automatic parameter measurement tool to extract acoustic parameters from spectrograms (FFT length = 256, frequency resolution = 31 Hz, temporal resolution  = 16 ms (overlap = 50%), window type  =  Hamming). For each element we measured: (i) the initial peak of fundamental frequency (defined as 'start F0'), (ii) the final peak of fundamental frequency (end F0) and (iii) the maximum peak of fundamental frequency (max F0). In addition, we calculated three temporal measures: (iv) duration (in seconds) of each element from the initial to the final F0, (v) duration (in seconds) between consecutive elements, and finally (vi) the temporal location (in seconds) of max F0 divided by the element duration (Fig. 1). Depending on the background noise we used a flexible threshold (ranging between −5 and −20 dB, mean value: 12.8) to distinguish between noise and signal. We combined the frequency measurements per call element to characterize changes at the call level. Beside mean values per element, we also included maximum of a call and variation within a call to account for variability between call elements. Together with call duration we had 22 acoustic parameters to characterize the gibbon calls in frequency and temporal domain (Table 2). For the 14 animals included into the acoustic analysis, we recorded a total of 48 songs, 784 calls and 3,993 elements.

**Table 2:** Results of the Factor Analysis (FA) and transformations applied.

	*Parameters*	*Transformation*	*Factors*					
			*1*	*2*	*3*	*4*	*5*	*6*
	call duration [s]	log(x)	−0.08	0.03	0.04	−0.05	**0.89**	0.03
**Mean**	element duration [s]		0.13	**0.64**	−0.26	0.25	0.24	−0.41
	interval duration [s]	sqrt(x-min(x))	0.06	0.43	−0.17	0.12	**0.63**	−0.47
	start F0 [Hz]		**0.68**	0.01	−0.07	0.04	−0.20	**0.58**
	end F0 [Hz]		**0.88**	−0.12	0.08	0.11	−0.17	0.17
	mean F0 [Hz]		**0.95**	0.05	0.06	0.03	−0.05	0.12
	max F0 [Hz]		**0.93**	0.11	−0.06	−0.02	0.07	0.09
	location of max F0 [s]	sqrt(x)	0.16	0.19	−0.20	**0.77**	0.03	−0.38
**Maximum**	element duration [s]		−0.07	**0.89**	−0.01	0.07	0.22	−0.04
	interval duration [s]	log(x)	−0.03	**0.52**	−0.02	0.01	**0.78**	−0.06
	start F0 [Hz]		0.44	−0.05	0.12	0.02	0.08	**0.81**
	end F0 [Hz]	sqrt(x-min(x))	**0.71**	−0.06	**0.51**	−0.01	−0.01	0.17
	mean F0 [Hz]	sqrt(x-min(x))	**0.69**	−0.01	**0.54**	0.06	0.01	0.13
	max F0 [Hz]		**0.69**	−0.09	0.40	−0.08	0.23	0.13
	location of max F0 [s]	sqrt(x)	−0.01	0.04	0.03	**0.95**	0.08	0.10
**Variation**	element duration [s]	sqrt(x)	−0.03	**0.94**	−0.01	0.07	−0.02	0.06
	interval duration [s]	log(x)	0.04	**0.66**	−0.02	0.06	**0.55**	0.13
	start F0 [Hz]	sqrt(x)	0.35	0.05	0.16	0.05	−0.03	**0.82**
	end F0 [Hz]	sqrt(x)	0.41	0.15	**0.69**	−0.11	−0.10	0.13
	mean F0 [Hz]	sqrt(x)	0.06	−0.02	**0.88**	0.07	−0.12	0.07
	max F0 [Hz]	sqrt(x)	0.05	−0.18	**0.71**	−0.07	0.13	0.05
	location of max F0 [s]	sqrt(x)	−0.03	0.10	0.04	**0.94**	−0.10	0.12
	Eigenvalues		6.62	4.45	2.48	1.84	1.50	1.23
	variance explained [%]		30.08	20.22	11.30	8.37	6.81	5.59

Indicated are the loadings of the acoustic parameters on the six derived factors (absolute loadings ≥0.5 are highlighted in boldface), Eigenvalues and percent variance explained by the factors. Mean  =  mean of calls' elements; Maximum  =  maximum value of calls' elements; Variation  =  variation of elements within a call.

### Statistical analysis

#### Factor analysis

To remove redundancy between the acoustic parameters we first ran a Factor Analysis (FA) on parameters derived from calls. This approach was justified as indicated by large correlations between the acoustic parameters, Bartlet's test of sphericity (χ^2^ = 30707, df  = 231; *P*<0.001) and the Kaiser-Meier-Olkin measure of sampling adequacy (0.711 [68]). Before running the FA, and in order to achieve an approximately symmetrical distribution, we checked the distribution of each of the 22 acoustic parameters and transformed variables when required (Table 2). The FA was run with varimax rotation, and we used the regression method to obtain scores for each of the factors derived. In the subsequent analyses we used the derived factor scores as measures of the acoustic properties of the songs recorded.

#### Inter-individuality

We used a Discriminant Function Analysis (DFA) to test for differences between calls of different individuals, and a permuted DFA to account for non-independence of calls recorded at the same day (pDFA [69]). Moreover, since vocal recordings of the same individual were sometimes collected during the same days, we also permuted calls day-wise between subjects. The DFA included a total of 10 individuals for which at least 23 calls were recorded (i.e., at least one more than the number of the acoustic parameters: [*N* = 22]). Prior to the DFA, we transformed variables as described above (Table 2). The total number of calls included in this analysis was 647.

To derive the discriminant functions and to balance the individual contribution, we used 23 randomly selected calls per individual, while to reduce the impact of any random selection we ran 100 random selections and averaged the results. *P*-values of the DFA were based on cross-validated calls and determined using 1,000 permutations into which the original data were included as one permutation. The pDFA was conducted in R using a script written by one of us (RM). To estimate the contribution of the individual acoustic parameters to the discriminability between males, we ran a DFA in SPSS (version 15) including all calls.

#### Relationship between acoustic parameters, socio-demographic features and fecal androgens

To investigate whether the acoustic structure of the call parameters (i.e., the factor scores derived from the FA) varied according to socio demographic features (i.e., social organization, social status and age) and male androgen levels, we used General Linear Mixed Models (GLMM [70]). We ran six separate models, each with one of the six factor scores describing the acoustic features of the calls as the response. We included into these models male status (pair-living male; primary male in uni-female/multimale units; secondary male in uni-female/multimale units), age (adult; senior) and fecal androgen level (see below) as fixed effects while group, subject identity, date (nested with subject) and song were included as random effects. To test whether variation in call parameters due to varying androgen levels was happening at the level of between subjects variation (i.e., effects of androgen being largely a function of overall differences between subjects with regard to their average androgen levels) and/or within subjects (i.e., call parameters varying as a functions of short term variations of androgen levels within subjects) we used within subjects centering [71]. More precisely, male androgen levels were represented in the models by two terms: one being the average androgen level per subject and one being the actual androgen values, centered to a mean of zero per subject (by subtracting from each value the mean androgen level of the respective subject). To control the possibility that the effect of varying androgen levels on the acoustic structure of the calls varied between males we also included a random slope component of (within subjects centerd) androgen level within males [72].

Due to the presumed time lag for metabolites excretion into feces in gibbons [57], [73], we considered day 3 after the vocal recording to be the day at which the fecal sample best reflects the androgen level at the day of recording. Thus, as a measure of androgen level we used values from fecal sample collected closest to this optimal day, whereby we considered only fecal samples which were collected between the day of recording and 7 days later. When several samples fulfilled this criterion we averaged the values. In total, we had 295 samples collected at the optimal day, 62 and 64 collected at the day of recording or the next day, respectively, and 3, 5, 22, 52 at days 4 to 7 after the recordings. Prior to the analysis, we z-transformed values of fecal androgen levels to a mean of zero and a standard deviation of one. We included only one type of song, male solos, and only adult males (we excluded subadults from the model), leaving to a total of 503 calls from 34 songs recorded on 24 days from 13 adult animals out of 10 groups.

The models were fitted using Gaussian error function and identity link. We checked for the assumptions of normally distributed and homogenous residuals by visually inspecting histograms and qq-plots of the residuals as well as residuals plotted against fitted values. None of these indicated severe deviations from these assumptions (assumptions were checked only after the autocorrelation term had been included; see below). Estimating the significance of fixed effects in mixed models is controversial [74]. Here we estimated *P*-values using Markov-chain Monte-Carlo (MCMC) analysis, presumably the most reliable method currently available [70].

Song structure was likely to show temporal autocorrelation (i.e., calls recorded closer to one another in time being more similar to one another than those recorded more distantly). Such temporal autocorrelation may lead to non-independent residuals and could potentially greatly devalue the validity of the model. Hence, we incorporated autocorrelation into the model by first running the full model (as described above) and retrieving the residuals from it. Subsequently, and separately for each data point, we calculated a weighted average of the residuals of all other data points from the same male with the weight of the residuals being proportionate to their time lag to the specific data point. The resulting variable, 'autocorrelation term', was then included as an additional fixed effect. The weight function had the shape of a Gaussian function with a mean at a time lag equal to zero. Its standard deviation was determined such that the likelihood of the full model with the autocorrelation term included was maximized. Time was measured on a continuous scale considering the actual time each particular call began. To achieve an easy and interpretable estimate we z-transformed the autocorrelation term to a mean of zero and standard deviation of one before including it in the model.

We tested for model stability, by excluding subjects one by one and comparing the estimates. This showed instability issues in some of them, but given the non significance of most of the estimates, this result could be expected. In fact, the only term that remained significant after the correction for multiple testing showed little variations in the revealed *P*-values.

Since testing the impact of fixed effects (fecal androgen level, social organization, social status and age) on the six factors required accounting for multiple testing. We hence applied Simes' method [75] for this purpose.

The FA and a single DFA were run in SPSS (15.0). All other analyses were conducted in R (2.15.2 [76]). GLMMs were calculated using the function lmer of the R package lme4 [77], MCMC *P*-values were derived using the function pvals.fnc of the R package languageR [78], the pDFA was based on the function lda of the R-package MASS [79], and the autocorrelation term was derived using a self-written function.

## Results

### Factor analysis

The FA revealed six factors with Eigenvalues >1 together explaining 82.4% of the total variance (Table 2). Based on the loadings of acoustic parameters on the rotated components we were able to characterize and label these factors.

All acoustic parameters describing the element pitch showed high loadings on Factor 1 (pitch). Factor 2 (element duration) showed high loadings of parameters describing mean and maximum duration of elements. Acoustic parameters which showed high loadings on Factor 3 (pitch variation) described the variation in F0 of elements. The three high loadings on Factor 4 (location of max F0) described mean, maximum and variation of the location of maximum F0. Total call duration loaded exclusively on Factor 5 (call duration) which, in addition, showed stronger loadings of acoustic parameters describing interval duration between elements. Factor 6 (start F0) showed only high loadings of parameters describing the start F0.

### Inter-individual differences

**Figure 2:**
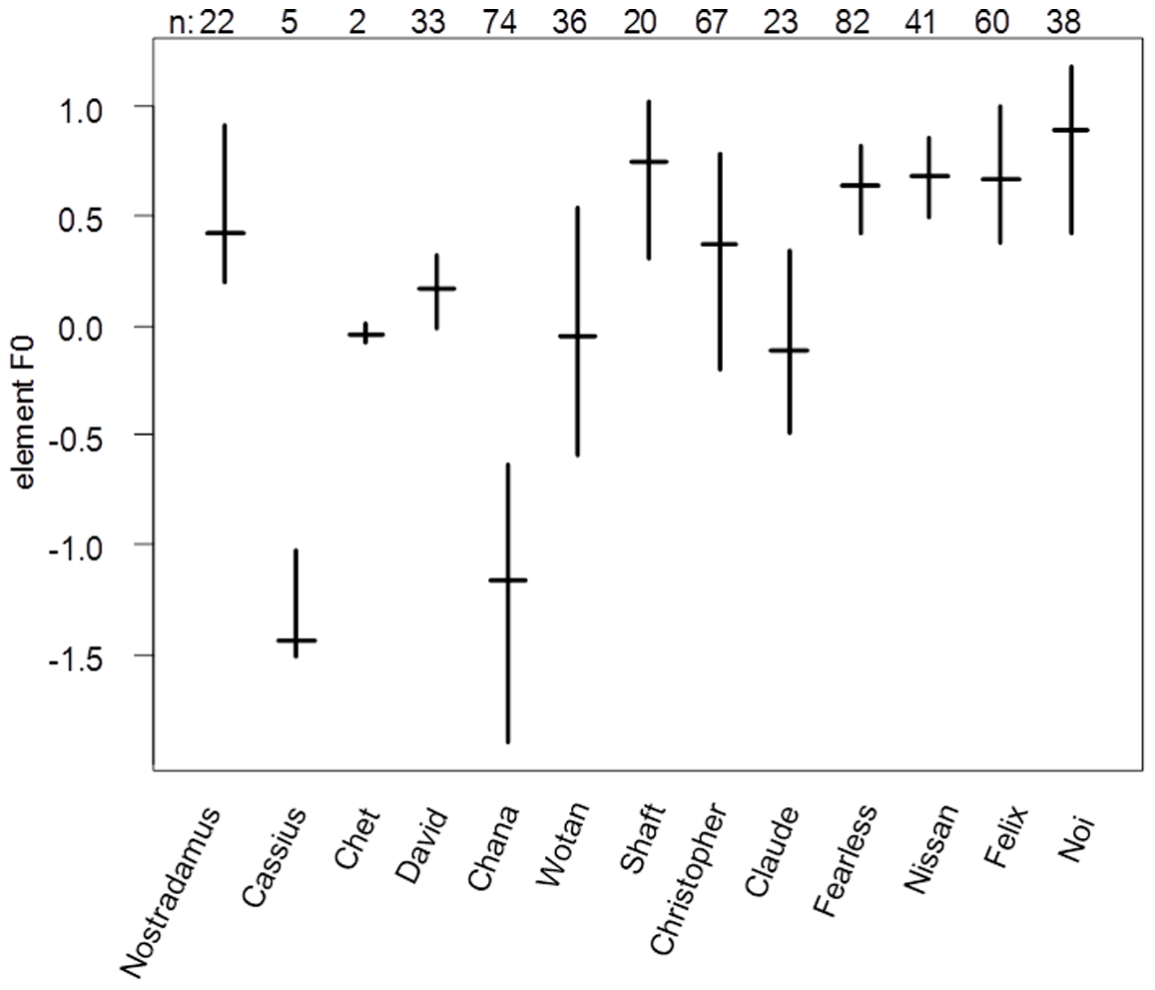
Individual differences expressed as factor score values representing elements' fundamental frequency (F0) of 13 adult male gibbons residing at Khao Yai National Park, Thailand. Full names of gibbons recorded are reported at the x-axis as well as total number of calls used in the analysis on top of the graph. Males are represented in order of increasing mean androgen levels. Indicated are medians (horizontal lines) and quartiles (vertical lines).

The pDFA revealed that calls differed between individuals (average percentage of correctly assigned cross classified calls: 74.6%, chance level  = 10%, *P* = 0.001; Fig. 2). Running a DFA on all calls revealed two discriminant functions with Eigenvalues >1. Variables with high absolute loadings (≥0.5) on any of the first two discriminant functions were (i) maximum element duration, (ii) variation in element duration and (iii) maximum of mean F0.

### Androgens, social status and age on gibbon calls

We found a clear link between fecal androgen levels and Factor 1 'element F0' (Table 3). Gibbons with higher average androgen levels produced calls having call elements with significantly higher pitch (Factor 1; Table 3; *see* also Appendix, Table S1). None of the other acoustics properties tested co-varied with androgen levels.

**Table 3:** Correlations between fecal androgen level, age, social status and call structure (estimates derived from GLMMs).

*Factors*	*Description*	*Androgen* 1	*Androgen* 2	*Age*	*Social status*
1	element F0	**<0.001↑**	0.096	0.843	0.14
2	element duration	0.366	0.33	0.747	0.14
3	F0 variation	0.192	0.348	0.843	0.92
4	location of max F0	0.366	0.509	0.843	0.14
5	call duration	0.499	0.278	**0.048 A>S**	0.221
6	start F0	0.571	0.084	0.843	0.92

1 androgen variation **between** subjects (i.e., effects of androgen as a function of differences between subjects with regard to their average androgen levels);

2 androgen variation **within** subjects (i.e., effects of androgen as a functions of short term variation of androgen levels within subjects).

*P*-values were corrected for multiple testing (Simes correction); significant differences were highlighted in boldface. The arrow shows the direction of changes for significant differences; A>S indicates that larger values were found in adult (A) than senior (S) males.

We also found that among adult males those of senior age had lower call duration (Factor 5; Table 3; Appendix, Table I). No obvious relation among any of the remaining call parameters considered was found between males belonging to different social status (Table 3).

Although only qualitative data were available, subadults (males already mature but still residing in their natal groups) presented interesting similarities to senior males (i.e., number of elements per call, number of call per song, start and maximum F0; Table 4). Indeed subadults differed from anybody else in call duration, duration of intervals between elements and element duration (Table 4).

**Table 4:** Median (quartiles in brackets) and range values (minimum and maximum) of acoustic parameters of male gibbon songs assessed in three age classes.

	INDIVIDUALS' AGE					
	*Subadult*		*Adult*		*Senior*	
*Acoustic parameters*	median	range	median	range	median	range
call duration [s]	10.3 (8.1, 12.8)	4.4–56.5	8.4(6.8, 10.6)	1.9–201.6	8.3 (7.2, 9.5)	1.7–29.8
number of elements	8 (6, 9)	4–50	9 (7, 12)	3–127	9 (8, 10)	1–33
element duration [s]	0.46 (0.20, 0.73)	0.01–1.73	0.27 (0.14, 0.58)	0.01–2.09	0.24 (0.13, 0.58)	0.02–1.73
interval duration [s]	0.88 (0.50, 1.24)	0.06–4.47	0.54 (0.32, 1.01)	0.04–4.34	0.46 (0.29, 0.97)	0.09–2.74
start F0 [Hz]	680 (640, 760)	530–1460	700 (620, 850)	390–1540	670 (600, 870)	10–1530
end F0 [Hz]	920 (820, 1040)	600–1700	950 (810, 1090)	480–1950	960 (820, 1100)	430–1570
max F0 [Hz]	930 (870, 1010)	640–1430	950 (840, 1040)	530–1480	930 (810, 1040)	500–1400
location of max F0 [s]	1040 (950, 1170)	710, 1900	1070 (930, 1210)	540–1950	1120 (960, 1210)	510–1680
number of elements	*N* = 2476		*N* = 5557		*N* = 2344	
number of calls	*N* = 259		*N* = 533		*N* = 257	

## Discussion

Our study aimed to investigate wild white-handed male gibbon solo songs with respect to individuality, hormonal underpinning and relationship to socio-demographic features such as social status and age. First, we confirm that male gibbon songs exhibit significant differences among individuals and such variation is expressed in terms of song characteristics. Individual differences are common in a variety of other primate vocalizations, including gibbons (male songs [54]; female songs [80]), baboons [81], [82], and chimpanzees [83], as well as in other animal species [84]–[86]. Many playback studies have shown that listeners can use this information to distinguish between group members [4], [87], [88].

Our results show a highly significant correlation between androgen levels and vocal pitch. Males with higher androgen levels produced elements having higher pitch. The relationship found between male androgen levels and their vocal parameters shows the opposite of what is known about the influence of testosterone on the male voice in humans [21], [89]. It is known that during puberty, elevated testosterone may act through androgen receptors on vocal folds and causes their growth [90], [91]. By lengthening and thickening the vocal folds, fundamental frequency becomes lower [92] and, as a consequence, men vocalize with lower pitch, approximately half of the F0 in women [93]. However, in studies of non-human primate vocalizations, data from two studies on chacma baboons indirectly suggest a positive relationship between male testosterone levels and vocal pitch [23], [24]. While running around or leaping through the trees, male chacma baboons give so-called 'wahoos' which are loud and highly costly calls. Fischer and colleagues [4] showed that high ranking males produce 'wahoos' with higher F0 and longer 'hoo' elements than low ranking males. When males fall in rank the 'hoo' syllables become shorter and F0 declines. In a subsequent study, measuring testosterone levels of chacma baboon males, it has been shown that high ranks are strongly correlated with higher testosterone levels and that with a decline in rank also testosterone levels decrease [23], [24]. Thus, although indirectly, these two studies strongly suggest that in chacma baboons an increase in testosterone is likely associated with an increase in F0, as observed in male gibbons of this study. Differently from chacma baboons, white-handed gibbons do not show an increase in element duration, but a significant increase in call pitch. Another study on male loud call characteristics of Thomas langurs (*Presbytis thomasi*) revealed an increase in tonal units and duration with increasing testosterone levels [94]. To our knowledge, those are the only studies of non-human primates which focused on a direct relation between androgen levels and vocal structure. Although our data and those of others are correlational, one possible interpretation is that androgen levels lead to a higher motivation to sing which may trigger structural changes in vocalizations, as it has been found in complex hyrax (*Procavia capensis*) songs [95]. Independent of the proximate mechanism, it is likely that group members use such structural differences to assess motivation and status of the caller.

Androgen receptors have been shown in laryngeal muscles and connective tissue of vocal folds as well as in the brain [34], [35]. Thus, it could be that higher androgen levels affect vocal folds and change the vibration characteristics although the excitation frequency remains the same. Chances in laryngeal muscles and connective tissue via androgen receptors are reversible to some extent and therefore in accordance with the observed reversed pattern for the relation between F0, androgen and age. In addition, the relations between vocal fold morphology and mechanical properties studied in several species can support the hypothesis that differences in mechanical properties can explain our observed vocal differences [96], [97]. However, it is also possible that elevated androgen levels could lead to a higher motivation to call. This higher motivation could lead to calls having higher amplitude and thus an increase in call pitch [98], [99]. All in all, these studies on male call structure in relation to androgen levels suggest that male vocal signals in gibbons are consistent with an effect of androgens and hence could function as a reliable signal of male competitive abilities.

Socio-demographic features did not seem to have any impact on song parameters, except for age. Although previous findings have revealed differences in androgen levels among males living in pairs than those living in unifemale/multimale units [59], no obvious differences were found in vocal signals between males living under different social conditions. Subadult and juveniles do more likely show high variation in androgens levels compared to adults; however, we did not consider them in the analysis. Thus, age appeared to have some sort of effect on vocal production also in a rather restricted narrow androgen concentration range (among adults of different age) when vocal anatomy maturation was certainly already completed. Assuming that singing is a costly signal difficult to produce and which may reflect relative quality of individuals, only physically fit males should be able to perform song for a longer period [100]. A comparison of call duration of senior and adult males showed that adult males sang longer than senior ones. Such finding, combined with the result that males having higher androgens sang with higher pitch voice, suggests that solo songs may potentially provide honest signals of male quality important in mate choice [101].

As in other animal species, male vocalizations could also help to estimate male fighting ability without engaging in direct contest for both mate partner and territory [102]. By identifying individuals' age, males should attempt to assess asymmetries in fighting ability before engaging in escalated and potentially costly fights [103]–[105]. However, since the type of song examined were not given during the context of group encounters, but in the early morning when no encounters took place, we can speculate that they could likely function in assessing more male quality rather than territorial defense.

## Supporting Information

Table S1 Results of the GLMMs with factor scores as the responses, and androgen, age, social status as predictors. The models account for androgen levels using two fixed effects, one accounting for varying androgen levels between subjects (average androgen levels per male) and one for the within subjects variation of androgen levels (androgen levels centered to a mean of zero per subject). Subadults were excluded from the data analyzed. Note that the *P*-values are not corrected for multiple testing.
(DOC)
