# Supplementary material for: Cues to Androgens and Quality in Male Gibbon Songs
Source: PLoS One. 2013 Dec 18;8(12):e82748. doi: 10.1371/journal.pone.0082748 (PMC3867390; doi:10.1371/journal.pone.0082748)
Supplement: Table S1 — Results of the GLMMs with factor scores as the responses, and androgen, age, social status as predictors. The models account for androgen levels using two fixed effects, one accounting for varying androgen levels between subjects (average androgen levels per male) and one for the within subjects variation of androgen levels (androgen levels centered to a mean of zero per subject). Subadults were excluded from the data analyzed. Note that the P-values are not corrected for multiple testing. (DOC) [file pone.0082748.s001.doc]

*Supporting Information*

**Table S1.** Results of the GLMMs with factor scores as the responses, and androgen, age, social status as predictors. The models account for androgen levels using two fixed effects, one accounting for varying androgen levels between subjects (average androgen levels per male) and one for the within subjects variation of androgen levels (androgen levels centered to a mean of zero per subject). Subadults were excluded from the data analyzed. Note that the *P*-values are not corrected for multiple testing.

|  |  |  | | |
| --- | --- | --- | --- | --- |
| **Factors** | **Predictor** | **Estimate** | **SE** | **P**_MCMC_ |
| *Factor 1* | intercept | 0.962 | 0.325 | (5) |
|  | androgen (within)^(1)^ | 0.157 | 0.094 | 0.032 |
|  | androgen (between)^(2)^ | 0.526 | 0.111 | 0.000 |
|  | age^(3)^ | 0.029 | 0.252 | 0.842 |
|  | status (multimale / secondary)^(4)^ | -1.046 | 0.383 | 0.026 |
|  | status (pair living)^(4)^ | -0.852 | 0.383 |  |
|  | autocorrelation term | 0.250 | 0.020 | <0.001 |
| *Factor 2* | intercept | -0.810 | 0.465 | (5) |
|  | androgen (within)^(1)^ | 0.141 | 0.084 | 0.220 |
|  | androgen (between)^(2)^ | 0.164 | 0.162 | 0.228 |
|  | age^(3)^ | 0.238 | 0.372 | 0.249 |
|  | status (multimale / secondary)^(4)^ | 0.836 | 0.566 | 0.070 |
|  | status (pair living)^(4)^ | 1.012 | 0.538 |  |
|  | autocorrelation term | 0.170 | 0.028 | <0.001 |
| *Factor 3* | intercept | -0.158 | 0.196 | (5) |
|  | androgen (within)^(1)^ | 0.068 | 0.065 | 0.290 |
|  | androgen (between)^(2)^ | 0.200 | 0.079 | 0.064 |
|  | age^(3)^ | -0.048 | 0.163 | 0.829 |
|  | status (multimale / secondary)^(4)^ | -0.078 | 0.236 | 0.792 |
|  | status (pair living)^(4)^ | -0.119 | 0.226 |  |
|  | autocorrelation term | 0.059 | 0.029 | 0.046 |
| *Factor 4* | intercept | 0.732 | 0.351 | (5) |
|  | androgen (within)^(1)^ | -0.073 | 0.127 | 0.509 |
|  | androgen (between)^(2)^ | 0.161 | 0.126 | 0.244 |
|  | age^(3)^ | 0.012 | 0.275 | 0.798 |
|  | status (multimale / secondary)^(4)^ | -1.111 | 0.416 | 0.063 |
|  | status (pair living)^(4)^ | -0.619 | 0.411 |  |
|  | autocorrelation term | 0.175 | 0.028 | <0.001 |
| *Factor 5* | intercept | -0.204 | 0.434 | (5) |
|  | androgen (within)^(1)^ | 0.213 | 0.125 | 0.139 |
|  | androgen (between)^(2)^ | 0.182 | 0.148 | 0.416 |
|  | age^(3)^ | -0.809 | 0.337 | 0.008 |
|  | status (multimale / secondary)^(4)^ | 0.642 | 0.512 | 0.147 |
|  | status (pair living)^(4)^ | 0.834 | 0.511 |  |
|  | autocorrelation term | 0.068 | 0.032 | 0.017 |
| *Factor 6* | intercept | -0.105 | 0.341 | (5) |
|  | androgen (within)^(1)^ | 0.388 | 0.146 | 0.014 |
|  | androgen (between)^(2)^ | 0.062 | 0.128 | 0.571 |
|  | age^(3)^ | 0.068 | 0.268 | 0.843 |
|  | status (multimale / secondary)^(4)^ | 0.226 | 0.405 | 0.920 |
|  | status (pair living)^(4)^ | 0.248 | 0.396 |  |
|  | autocorrelation term | 0.065 | 0.034 | 0.059 |

^(1)^: androgen levels centered to a mean of zero per subject; ^(2)^: mean androgen level per subject; ^(3)^: adult = 0, senior = 1; ^(4)^: indicated are the estimated differences (and associated standard errors) between the two dummy coded levels of the factor status (secondary male in unifemale/multimale, and primary males in unifemale/unimale groups, respectively) and primary males in unifemale/unimale groups; the P-value indicated refers to the overall test of the effect of status; ^(5)^: not indicated because of having no meaningful interpretation.
